# Incidence and prevalence of lower extremity tendinopathy in a Dutch general practice population: a cross sectional study

**DOI:** 10.1186/s12891-016-0885-2

**Published:** 2016-01-13

**Authors:** Iris Sophie Albers, Johannes Zwerver, Ronald Leo Diercks, Janny Hendrika Dekker, Inge Van den Akker-Scheek

**Affiliations:** Center for Sports Medicine, University of Groningen, University Medical Center Groningen, Hanzeplein 1, 9700 RB Groningen, The Netherlands; Department of General Practice, University of Groningen, University Medical Center Groningen, Groningen, The Netherlands

**Keywords:** Epidemiology, Patellar, Tendinitis, Tendonitis, Tendon, Fasciitis, Heel, Spur

## Abstract

**Background:**

Lower extremity tendinopathy is a common sports injury, but it can also affect non-athletes. Because tendinopathy is difficult to treat and has negative effects on the ability to work and quality of life, development of preventive interventions is important. The first step in the Van Mechelen prevention model is to determine the extent of the problem. The primary aim of this study was to determine the incidence and prevalence of lower extremity tendinopathy in a Dutch general practice population. The secondary aim was to investigate possible associated factors.

**Methods:**

A cross-sectional study was performed in a Dutch general practice. Using International Classification of Primary Care codes, the electronic patient files were searched to identify cases of adductor tendinopathy, greater trochanteric pain syndrome, jumper’s knee, Achilles tendinopathy, and plantar fasciopathy in 2012. The tendinopathy patients were compared to the general practice population regarding age, gender, use of medication, and comorbidity using 95 % confidence intervals.

**Results:**

The prevalence and incidence rates of lower extremity tendinopathy found in this study were 11.83 and 10.52 per 1000 person-years. Lower extremity tendinopathy was more prevalent among older patients. No differences between tendinopathy patients and the general practice population were found regarding gender, use of medication, or comorbidity.

**Conclusions:**

In this cross-sectional study in a Dutch general practice, the prevalence and incidence rates of lower extremity tendinopathy were 11.83 and 10.52 per 1000 person-years. Lower extremity tendinopathy deserves a higher place in locomotor system research to develop preventive interventions.

## Background

Lower extremity tendinopathy is a common injury in athletes, but it can also develop in non-athletes [1, 2]. Because tendinopathy can be difficult to treat and might have negative effects on not only **s**ports participation, but also on the ability to work and quality of life [3, 4], development of preventive interventions is of utmost importance.

The first step in the Van Mechelen model for prevention is to determine the extent of the problem [5]. The incidence and prevalence of lower extremity tendinopathy among specific sports populations have been established [6–9]. However, these numbers are largely lacking for the general practice population. The epidemiology of only two lower extremity tendinopathies – Achilles tendinopathy and greater trochanteric pain syndrome – have been studied in Dutch general practice populations [2, 10]. Therefore, the primary aim of this study was to determine the incidence and prevalence of lower extremity tendinopathy among a population visiting a Dutch general practice.

The second step in the Van Mechelen model is to determine risk factors. In previous studies, several factors have been investigated for a possible association with the development of tendinopathy. Aside from increasing age [11, 12], gender differences [13], and use of medication (corticosteroids, fluoroquinolones, and statins) [14–16], co-morbidities that have been linked to tendinopathy are hypertension, dyslipidaemia, and diabetes mellitus [17]. The secondary aim of this study was to investigate whether in this general practice population an association between tendinopathy and gender, age, use of medication, or co-morbidities could be found.

## Methods

To determine the incidence and prevalence of lower extremity tendinopathy in a Dutch general practice population, a cross-sectional study was performed at the Academic General Practice Groningen. This group practice of eight general practitioners with more than 10,500 registered patients is part of the morbidity and medication Registration Network Groningen (RNG), which systematically collects patient data in a longitudinal database. The practice was visited to identify cases of lower extremity tendinopathy in the electronic patient files. The following tendinopathies were investigated: adductor tendinopathy, greater trochanteric pain syndrome, jumper’s knee, Achilles tendinopathy, and plantar fasciopathy. Like other general practices in the Netherlands, the Academic General Practice Groningen uses International Classification of Primary Care (ICPC) codes [18] to document the reason of a patient’s visit. An ICPC code consists of a letter and two numbers, and represents a disease or a symptom. Because there are no specific ICPC codes for lower extremity tendinopathies, a combination of ICPC codes that the general practitioner could have used in the case of lower extremity tendinopathy was used in this study (Table 1). All patient contacts of the year 2012 that matched one of these ICPC codes were studied by ISA to identify cases of tendinopathy.

**Table 1:** ICPC codes and corresponding symptoms/diagnoses

Code	Symptom	Code	Diagnosis
L13	Symptoms/complaints hip	L99	Other disease(s) locomotor system
L14	Symptoms/complaints leg		
L15	Symptoms/complaints knee	L99.01	Bursitis
L16	Symptoms/complaints ankle	L99.02	Tendovaginitis/tendinitis
L17	Symptoms/complaints foot/toe	L99.08	Heel spur/plantar fasciitis
L29	Other/multiple symptoms/complaints locomotor system		

Because we investigated more than one tendinopathy, there was not a single set of inclusion criteria. Instead, we formulated the inclusion criteria per tendinopathy (Table 2). Patients with bilateral symptoms of one tendinopathy were scored as one case for that specific tendinopathy. Patients with symptoms of two or more different tendinopathies were scored as one case for each of the separate tendinopathies. Patients with tendon ruptures were excluded.

**Table 2:** Inclusion criteria per tendinopathy

Adductor related tendinopathy, one of the following	
- Diagnosis tendinopathy/tendinitis of the hip adductors	
- Pain in groin during adduction against resistance	
- Pain located at the insertion of the hip adductors on the pubic bone	
Greater trochanteric pain syndrome, one of the following	
- Diagnosis greater trochanteric pain syndrome/trochanteric bursitis	
- Pain on palpation of the greater trochanter	
Jumper’s knee, one of the following	
- Diagnosis jumper’s knee, tendinopathy/tendinitis of patellar tendon or quadriceps tendon	
- Pain located at the insertion of the quadriceps tendon on the patella	
- Pain located in the patellar tendon or its insertion on patella or tibial tuberosity	
Achilles tendinopathy, one of the following	
- Diagnosis tendinopathy/tendinitis of Achilles tendon	
- Pain located in Achilles tendon or its insertion on calcaneus	
Plantar fasciopathy, one of the following	
- Diagnosis plantar fasciitis/fasciopathy or heel spur	
- Pain in plantar fascia or its insertion on calcaneus	

To investigate possible associated factors, we documented age, gender, use of systemic corticosteroids, fluoroquinolones, or statins in the six months before diagnosis, and if the patient suffered from one of the following co-morbidities: diabetes mellitus (defined as ICPC code T90, T90.01, or T90.02), hypertension (defined as ICPC code K86 or K87), and dyslipidaemia (defined as ICPC code T93). We also documented the presence of overweight, defined as an ICPC code (T82 and T83 stand for obesity and overweight, respectively) or a remark about overweight by the general practitioner in the patient file. In addition, we documented the duration of symptoms before the patient visited the general practitioner, and the patient’s sports activities, if this information was available from the patient file.

The number of prevalent and incident cases was divided by the number of person-years in the practice population to determine a prevalence rate (PR) and incidence rate (IR) per 1000 person-years. We used the amount of person-years, because the population of a general practice is dynamic. Throughout the year, the population increases and decreases as a result of birth, death, and migration. The difference between incidence and prevalence was based on patient history. If there had been contacts for the same symptom in 2011, the patient was scored as a prevalent case only. If a patient had not visited the general practitioner for the same symptom in 2011, he or she was scored as both an incident and a prevalent case in 2012.

The tendinopathy patient data were compared to data of the general practice population that were obtained from the annual report of the RNG. We constructed 95 % confidence intervals to test for differences between the tendinopathy patients and the general practice population regarding gender, age, use of medication, and co-morbidities. If the confidence intervals overlapped, the difference was not considered statistically significant. If the confidence intervals did not overlap, the difference was considered statistically significant. SPSS version 22 was used to analyse the data.

## Ethics

The study protocol was reviewed and exempted from requiring ethics approval by the medical ethical committee of the University Medical Center Groningen (METc 2013/516).

Upon registration at the Academic General Practice, patients included in this study gave informed consent to the use of their anonymized data by the Registration Network Groningen.

## Results

Between January and April 2014, the Academic General Practice was visited to search the electronic patient files. In 2012, the Academic General Practice contained 10,651 person-years. The combination of ICPC codes provided 1091 hits in the electronic database. Based on the consultation reports, 126 prevalent cases of lower extremity tendinopathy were identified. Fourteen of these patients had visited the general practitioner in 2011 for the same symptoms. This led to a prevalence rate (PR) of 11.83 per 1000 person-years, and an incidence rate (IR) of 10.52 per 1000 person-years. The PR and IR per tendinopathy are shown in Table 3 (Table 3).

**Table 3:** Prevalence rate and incidence rate per tendinopathy

Tendinopathy	Prevalence	PR	Incidence	IR
Adductor related tendinopathy	13	1.22	12	1.13
Greater trochanteric pain syndrome	45	4.22	35	3.29
Jumper’s knee	17	1.60	17	1.60
Achilles tendinopathy	25	2.35	23	2.16
Plantar fasciopathy	26	2.44	25	2.34
Total	126	11.83	112	10.52

*PR* prevalence rate per 1000 person-years, *IR* incidence rate per 1000 person-years (practice population = 10,651 person-years)

The demographics of the tendinopathy patients and the practice population are shown in Table 4 (Table 4). There was no statistically significant difference in the percentages of men and women. The mean age of the tendinopathy patients was significantly higher than the mean age of the practice population.

**Table 4:** Patient characteristics tendinopathy patients vs. practice population [19]

	Tendinopathy patients (*n* = 126)	Practice population (*n* = 10,651)
Gender		
Male	41.3 % (95 % CI 32.6–50.0)	49 %
Female	58.7 % (95 % CI 50.0–67.4)	51 %
Mean age	46 years (95 % CI 43–49)*	36 years
Age categories		
0–17	4.0 % (95 % CI 0.5–7.4)*	16 %
18–44	42.1 % (95 % CI 33.3–50.8)	48 %
45–64	35.7 % (95 % CI 27.2–44.2)*	26 %
65+	18.3 % (95 % CI 11.4–25.1)*	9 %

95 % CI = 95 % confidence interval, *significant for *p* ≤ 0.05

Twelve of the 126 tendinopathy patients had diabetes (9.5 %; 95 % CI 4.3–14.6 %). This percentage was twice as high as the percentage of patients with diabetes in the general practice population (4.8 %) [19], but the difference was not statistically significant. Of the tendinopathy patients, 22 had hypertension. The general practitioners made a distinction between essential hypertension (ICPC code K86) and hypertension with organ damage (ICPC code K87). Eighteen tendinopathy patients had essential hypertension. This percentage (14.3 %; 95 % CI 8.1–20.5 %) was not significantly higher than the percentage in the general practice population (11.4 %) [19]. Ten tendinopathy patients had dyslipidaemia (7.9 %).

Fifteen tendinopathy patients used statins (11.9 %; 95 % CI 6.2–16.6 %). This percentage was not significantly higher than the percentage of patients using antilipaemic medication in the total practice population (7.3 %) [19]. Three tendinopathy patients were treated with systemic corticosteroids (2.4 %) and one tendinopathy patient was treated with fluoroquinolones (0.7 %) before the start of the symptoms.

Obesity or overweight were reported in fourteen cases (11.1 %), but no exact data concerning BMI were available. A relationship with sports activities was described in 37 cases (29.4 %). The duration of symptoms before visiting the general practitioner was reported in 40 cases and ranged from one day to six years with a mean of 32 weeks.

## Discussion

Lower extremity tendinopathy has an incidence rate of 10.52 per 1000 person-years and a prevalence rate of 11.83 per 1000 person-years in a Dutch general practice population. The mean age of the tendinopathy patients was higher than the mean age of the general practice population. There were no statistically significant differences between the tendinopathy patients and the general practice population regarding gender, use of medication, or co-morbidities.

This is the first study to investigate the incidence and prevalence of adductor related tendinopathy, jumper’s knee, and plantar fasciopathy in a general practice population. The incidence and prevalence for Achilles tendinopathy in a general practice population has been studied once before; our results are similar to the numbers found by De Jonge et al. in the Netherlands in 2011 [2]. The incidence of greater trochanteric pain syndrome was higher than the incidence of 1.8 per 1000 per year found by Lievense et al. [10]. However, in their study only 54 % of the originally selected patients responded to the questionnaire designed to verify the patients’ symptoms, which could introduce a bias and might explain the difference with our results.

To put the numbers into perspective, the total incidence and prevalence of locomotor system symptoms seen by Dutch general practitioners are 267 and 397 per 1000 persons per year, respectively [20]. The numbers found in our study indicate that the incidence of lower extremity tendinopathy represents 3.0 % of all new locomotor system symptoms seen by general practitioners. In addition, the incidence found for tendinopathy in the current study was higher than the incidence of osteoarthritis in the Dutch general practice population in 2012 (8.4 per 1000 person-years) [21]. While prevention of osteoarthritis is already high on international research agendas [22], the prevention of tendinopathy has received little attention. The negative impact that tendinopathy can have on the ability to work and quality of life of patients calls for more research into preventive measures, especially because the treatment can be difficult [3, 4].

In the current study, the mean age of tendinopathy patients was significantly higher than the mean age of the general practice population. Earlier studies have found tendinopathy to be more prevalent among older people [11, 12]. The exact mechanism why older tendons seem to be more prone to damage remains to be elucidated. One theory attributes the effect to the accumulation of chronic repetitive damage over time [23]. Another hypothesis is that age-related changes in tendon structure and biomechanics could decrease the loading capacity and regenerative capacity of the tendons [24].

We found no significant difference in the percentages of men and women when we compared the tendinopathy patients to the practice population. Research among athletes shows different outcomes per tendinopathy. The male gender is considered a risk factor for jumper’s knee [13, 25]. Earlier, the male gender was found to be a risk factor for Achilles tendinopathy as well [26]. However, in athletes over 40 years of age, Longo et al. did not find a difference in risk between men and women [27]. Plantar fasciopathy does not seem to have a male or female predisposition [28].

Although in the current study the prevalence of diabetes among the tendinopathy patients was twice as high as the prevalence of diabetes in the general practice population, this difference was not statistically significant. This conflicts with earlier research, in which diabetes was found to be a risk factor for tendinopathy [29]. It is possible that our relatively small study population explains this difference in outcomes. There is evidence that diabetes can alter tendon structure, possibly through an excess of advanced glycation end products as a result of hyperglycaemia [17]. In this study, we did not look into the levels of blood glucose in the patients with diabetes. In general, Dutch patients who are being checked by their general practitioner or practice nurse are well regulated. However, we cannot exclude the possibility that poorly regulated or not yet diagnosed patients with diabetes were in the dataset. Gaida et al. have questioned whether the higher risk of tendinopathy associated with diabetes is caused by a disturbance in glucose metabolism per se. They proposed that the higher risk might have to be attributed to the overweight associated with diabetes [30, 31]. The influence of overweight on tendinopathy has been explained in two different ways. One of the theories is that the extra weight leads to an increased loading of the tendons. However, this theory does not explain why non-weight bearing tendons also more often develop tendinopathy in overweight persons [11]. Another theory is that lipocytes have an effect on tendon structure by their metabolic activity [30]. In a recent review on tendinopathy in athletes, Scott et al. state there is evidence for both a mechanical effect and a systemic effect, but the relative contributions of the two mechanisms should be further investigated [31]. In this study, no exact data on BMI of the tendinopathy patients were available.

There were no significant differences in the prevalence of hypertension between the tendinopathy patients and the practice population. Only few patients had dyslipidaemia, so we could not draw conclusions on the association between dyslipidaemia and tendinopathy. We did not have data on how well controlled the patients with hypertension and dyslipidaemia were. Future research could clarify whether developing tendinopathy is related to levels of control of lipids and tension.

Although the use of corticosteroids and fluorquinolones has been identified as a risk factor for developing tendinopathy [14, 15], in this study only few patients were treated with corticosteroids or fluoroquinolones before the start of the symptoms, so we could not draw conclusions on the association between the use of these medications and tendinopathy. The percentage of tendinopathy patients using statins was not significantly different from the percentage of people using antilipaemic drugs in the total practice population. Marie et al. investigated reports on statin-induced tendinous disorders and concluded that these complications are very rare, given the large number of statin prescriptions [16]. The mechanism behind these complications is unknown, but in a rat study De Oliveira et al. described an association between statin treatment and alterations in tendon metabolism [32].

Several factors could have influenced the outcomes of this study. An important factor is the help-seeking behavior of patients and the organization of (para)medical care in the Netherlands, which could have led to an underestimation of the actual incidence and prevalence of lower extremity tendinopathy in the general population. People with musculoskeletal problems do not always seek professional help. Picavet et al. state that half of the people with musculoskeletal complaints visit a health care professional, and 33 to 42 % of people visit a general practitioner for their complaints [33]. In addition, since 2006 patients do not need to be referred by their general practitioner to visit a physical therapist. Podotherapists are directly accessible since 2011, and in the studied time frame patients did not need to be referred by their general practitioner to visit a sports physician either. People with tendinopathy symptoms could therefore have gone directly to one of these healthcare professionals, instead of visiting their general practitioner. Another factor which could have influenced the outcomes, is the use of ICPC codes. Although a wide combination of ICPC codes was used to identify the cases, it is possible that some cases were missed. A general practitioner could have accidentally given the wrong ICPC code to a consult. In the current study, one case of Achilles tendinopathy was found under the ICPC code L15 (knee complaints).

A factor that could have led to an overestimation of the actual incidence and prevalence of lower extremity tendinopathy, is the inclusion of the greater trochanteric pain syndrome. Although most cases of greater trochanteric pain syndrome are caused by gluteal tendinopathy [34–36], there is a possibility that in some patients the pain was caused by e.g. primary trochanteric bursitis or referred pain from degenerative joint disease.

As this study focused on the epidemiology in primary care, we followed the clinical description and diagnosis as performed by the general practitioners. This entails that the diagnoses were not confirmed by medical imaging as general practitioners rarely order diagnostic imaging when a tendinopathy is suspected. As this was not the scope of this study, validation of these clinical diagnoses by adding additional investigations like medical imaging should be object of future research.

A strength of this study is that it provides insight into the incidence and prevalence of lower extremity tendinopathy among the general practice population, which was currently lacking. It also provides an easily applicable search strategy that allows to investigate multiple tendinopathies at the same time. Limitations of this study are the retrospective design and the relatively small study population, which might hinder extrapolation of the results. However, the numbers for Achilles tendinopathy in the current study were similar to those found by De Jonge et al., who collected data from different geographical locations in the Netherlands [2].

## Conclusions

Lower extremity tendinopathy has an incidence rate of 10.52 per 1000 person-years and a prevalence rate of 11.83 per 1000 person-years in a Dutch general practice population. In contrast to other research, in this general population sample the prevalence of tendinopathy was not higher in patients with diabetes or patients with hypertension. The mean age of the tendinopathy patients was higher than the mean age of the general practice population. In only 29.4 % of the cases, a relationship with sports was described. This indicates that tendinopathy is not only a sports injury, but is common in non-athletes as well. Lower extremity tendinopathy as common and bothersome condition deserves a higher place on the agenda of locomotor system research in order to develop more effective preventive interventions.

### Availability of data and materials

The anonymized data set can be requested through ISA.
